# Potential Transfer of Toxic Gluten from Biodegradable Tableware to Gluten-Free Foods: Implications for Individuals with Gluten-Related Disorders

**DOI:** 10.1021/acs.jafc.5c07516

**Published:** 2025-10-22

**Authors:** Carolina Sousa, Abel Heredia, Lucía de Arcos, Verónica Segura, Ángela Ruiz-Carnicer, Isabel Comino

**Affiliations:** Department of Microbiology and Parasitology, Faculty of Pharmacy, University of Seville, 41012 Seville, Spain

**Keywords:** biodegradable food-contact materials, gluten contamination, gluten migration, gluten related-disorders, celiac disease, food packaging safety, allergen

## Abstract

The increasing use of biodegradable food-contact materials poses a risk for individuals with gluten-related disorders, including celiac disease. Tableware manufactured from wheat or other cereal derivatives may retain gluten proteins; regulations do not mandate allergen labeling. This study evaluated gluten transfer from eight commercial biodegradable items to representative gluten-free foods under realistic conditions. Gluten was quantified in biodegradable tableware and food samples after contact, using monoclonal antibody-based assays (G12 and A1) which detect gluten immunogenic peptides (GIP), providing a sensitive measure of potential immunological risk. Only one wheat-derived dish contained gluten and transferred it into solid and liquid foods. Migration was greater in liquid foods, particularly emulsified systems. In several cases transferred gluten exceeded the 20 mg/kg threshold for gluten-free labeling. Heat and prolonged exposure increased transfer. These findings highlight a critical regulatory gap, underscoring the urgent need for mandatory allergen labeling on biodegradable tableware to protect vulnerable consumers.

## Graphical abstract

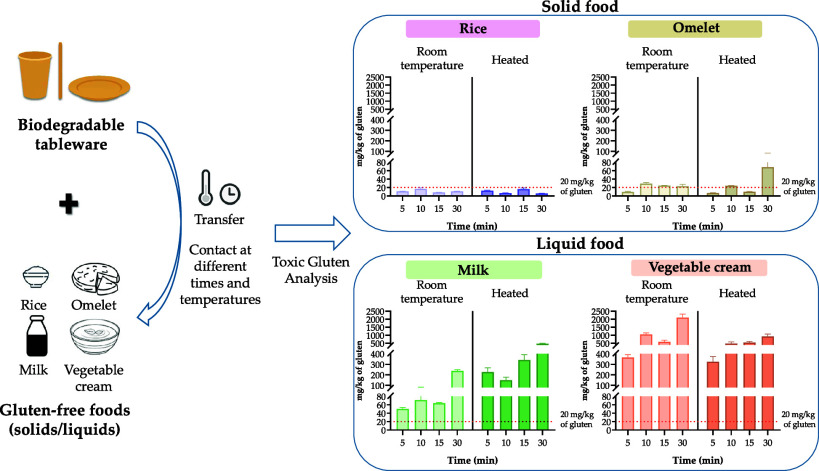

## 1 Introduction

Gluten is a complex mixture of proteins found in the endosperm of cereals, such as wheat, rye, barley, oats, and their derivatives. It has played a vital role in human diets for centuries, serving as a staple food across diverse cultures globally. The unique properties of gluten, characterized by its elasticity and cohesiveness, result from the interaction between its two main protein fractions, gliadin and glutenin. Gliadin, which is soluble in alcohol, contributes to the extensibility of gluten, allowing dough to stretch without tearing during shaping and proofing. Conversely, glutenin, which is insoluble in alcohol, provides strength and elasticity to the dough, facilitating gas retention and expansion during fermentation.^1,2^ These properties indicate that gluten is currently used not only in the food industry, but also in the manufacturing of medicines, cosmetics, and biopolymers. Despite its crucial role in food production, gluten is also associated with a spectrum of disorders that are prevalent worldwide. Gluten-related disorders (GRDs) include wheat allergy, nonceliac gluten sensitivity (NCGS), gluten ataxia, dermatitis herpetiformis, and celiac disease (CD).^3^

CD has been extensively studied and the role of gluten in its pathogenesis has been clearly identified. CD is a systemic disorder that results in chronic inflammatory enteropathy of the small intestine due to an inappropriate immune response to gluten in genetically predisposed individuals. The resistance of gluten to complete degradation by digestive enzymes and the subsequent generation of gluten immunogenic peptides (GIP), which are deamidated by tissue transglutaminase, are key events that trigger the immune response in CD. In addition to these intrinsic factors, various environmental influences—including viral infections, gut microbiota composition, and early life dietary exposures have also been proposed to modulate disease onset and severity.^4^ This response leads to inflammation, villous atrophy, and damage to the intestinal mucosa, impairing nutrient absorption and causing a wide range of gastrointestinal and extra-intestinal symptoms.^5,6^ Epidemiological studies suggest that approximately 1% of the general population is affected, although the actual prevalence may be higher due to underdiagnosis and asymptomatic cases.^7,8^ Currently, the cornerstone of GRDs management revolves around strict adherence to a lifelong gluten-free diet (GFD) that involves the complete avoidance of gluten-containing foods and ingredients. This dietary modification aims to alleviate symptoms, promote mucosal healing, and prevent long-term complications, such as malabsorption, nutritional deficiencies, osteoporosis, and increased risk of certain malignancies.^9^

Gluten-free foods can be classified into two main categories: natural and commercial. Natural gluten-free foods include whole unprocessed items such as fruits, vegetables, lean meats, poultry, fish, eggs, legumes, nuts, seeds, and certain grains such as rice, quinoa, and buckwheat. These foods are inherently gluten-free and serve as the foundational elements of a GFD. In contrast, commercial gluten-free products are manufactured as substitutes for traditional gluten-containing foods and encompass a wide range of items such as bread, pasta, cereals, snacks, and baked goods. These commercial alternatives are formulated using gluten-free ingredients and specialized processing techniques to mimic the taste, texture, and functionality of their gluten-containing counterparts, offering the patients with GRDs a convenient and accessible means of adhering to their dietary requirements while maintaining dietary variety.^10^

Stringent regulations exist in both Spain and the broader European Union (EU), to safeguard the dietary needs of patients with GRDs. Moreover, specific regulations governing gluten-free claims have been established to ensure the safety and reliability of gluten-free products. At the European level, the European Commission Regulation No. 41/2009 and No. 828/2014 have defined the criteria and threshold levels for labeling products as “gluten-free” setting the maximum permissible gluten content at 20 ppm (mg/kg) for gluten-free products.^11,12^ Moreover, EU Regulation No. 1169/2011 mandates the clear and accurate labeling of allergens, including gluten-containing ingredients, on food products.^13^ However, there are currently no regulations that establish gluten levels in other types of nonfood materials used to manufacture packaging, such as biopolymers.

With increasing concern over the long-term consequences of plastic waste on ecosystems and human health, there has been a growing emphasis on transitioning toward sustainable packaging solutions. This shift has been further propelled by the EU ban on certain single-use plastics, including plates, cutlery, straws, drink stirrers, and cups or containers made of expanded polystyrene, effective since July 2021 by the Directive (EU) 2019/904.^14^ Consequently, the adoption of biobased and biodegradable polymers derived from biomass as renewable resources has accelerated. Biodegradable packaging offers several advantages, including reduced reliance on fossil fuels, reduced carbon footprint, potential for organic decomposition, minimization of environmental contamination, and promoting of circularity within the packaging lifecycle.^15,16^

Biopolymers used in materials intended for food contact can be synthesized from various biomass sources, including polysaccharides such as alginate, carrageenan, chitosan, and pectin, as well as proteins derived from milk, egg, soy, and gluten. Wheat gluten is extracted from wheat flour by washing it with water during the starch extraction. Once dried to a powder, it maintains its viscoelastic, cohesive, and film-forming properties when rehydrated, making it particularly suitable for use in materials intended for food contact.^17^ However, there are several concerns for patients with GRDs because there is currently no regulation requiring the labeling of these allergens in biodegradable tableware, posing potential health risks. The raw materials used in the production of such materials are often unknown, as are the production processes and the risk of cross-contamination. If a material is unstable during use, it can easily break down and be inadvertently ingested. In individuals with CD or gluten sensitivity, trace amounts of gluten can trigger adverse immune responses, leading to severe health complications. Accurate detection and quantification of gluten contamination are essential to safeguard these vulnerable populations, ensuring the integrity of GFD and preventing inadvertent exposure.^18^

Research on the allergenic potential of materials intended for food contact and, more importantly, on the transfer of allergens from such materials into the final consumed food, is largely lacking.^19^ Therefore, this study aimed to investigate the transfer of gluten from biodegradable packaging into gluten-free foods to assess the risk of exposure in patients with GRDs.

## 2 Materials and Methods

### 2.1 Tableware Samples

Eight different types of biodegradable packaging were analyzed in this study. These included dishes, straws, and cups made of wheat and other potential organic gluten-containing materials from different manufacturers in the European Union, being all of them labeled as biodegradable. The composition of the tableware varied, as did their shape and specified instructions. All items were labeled as being made from wheat or similar byproducts (Table 1).

**Table 1:** Biodegradable Tableware Information^a^

code number	tableware	material	reusable	microwave-compatible	eatable
1	dish	palm leaf	yes	yes	no
2	dish	wheat pulp	nonspecified	yes	no
3	dish	wheat	nonspecified	yes	no
4	dish	wheat, coated with a plastic layer	yes	yes	no
5	dish	wheat	no	yes	yes
6	cup	wheat, coated with a plastic layer	yes	yes	no
7	straw	wheat	no	N.A.	no
8	straw	sugar cane	no	N.A.	no

a N.A., not applicable.

### 2.2 Food Samples

The foods used to study the gluten transfer from the tableware were two solid foods (omelet and instant rice) and two foods of liquid consistency (vegetable cream and milk). All were labeled as gluten-free foods. These items were selected because of their gluten-free nature, ensuring that any detected gluten presence originated from cross-contact rather than from inherent gluten content. Gluten content in each sample (0.5 g) was quantified to ensure the absence of gluten and validate its use as a gluten-free control substrate.

### 2.3 Sample Preparation and Extraction Methods for Gluten Analysis in Tableware and Foods

Prior to the experiments, all tableware (including dishes, cups, and straws) were analyzed to determine whether they contained gluten due to their composition. This analysis was crucial to ensure that any gluten detected during the experiments could be solely attributed to cross-contact with the tableware rather to the inherent gluten content. A previous extraction procedure was required to prepare the tableware samples for gluten quantification. The organic tableware was pulverized using the TissueLyser II (QIAGEN, Hilde, Germany), which simultaneously disrupts multiple samples through high-speed shaking in stainless steel tubes. The samples (0.5 g) were weighed and transferred individually to propylene tubes. They were then extracted with 5 mL of Universal gluten extraction solution (UGES) (Hygiena, Seville, Spain) followed by incubation at 50 °C in a water bath for 40 min.^20^ Finally, each suspension was centrifuged at 2500*g* for 10 min, and the supernatant was collected.

Samples (50 mL of milk or vegetable cream, and 50 g of rice or omelet) were weighed and individually placed in the selected dishes, cups, and straws. Cups and straws were tested exclusively with liquid samples. Experiments were conducted at different temperatures (room temperature, 20 ± 5 °C, and after microwave heating at 800 W for 1, 2, or 3 min) and at varying contact times (5, 10, 15, and 30 min). Continuous stirring with metal spoons was applied to simulate the estimated consumption period (Figure 1). For dishes and cups, only the food-contact surface was used, whereas straws were completely immersed. In microwaved samples, the heating time (1–3 min) was included as part of the total contact duration. After this contact with the tableware, solid foods were crushed, and 0.5 g of each of them (solid and liquid food samples) was weighed and individually transferred to polypropylene tubes (Jet Biofil, Elgin, IL, USA). They were then extracted with 5 mL of UGES (Hygiena Diagnóstica España S.L., Seville, Spain), followed by incubation at 50 °C in a water bath for 40 min. Finally, each suspension was centrifuged at 2500*g* for 10 min and the supernatant was collected.

**Figure 1:**
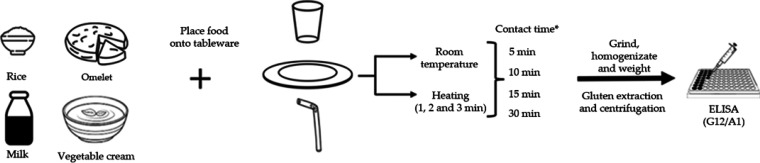
Experimental workflow for quantifying gluten transfer from tableware into food. *The microwave heating period (1–3 min) was included in the total contact time.

### 2.4 Gluten Quantification

The gluten concentration in the samples (tableware, food and food in contact with tableware) was quantified using the GlutenTox G12-A1 enzyme-linked immunosorbent assay (ELISA) Rapid Kit (Hygiena, Seville, Spain), which consists of a sandwich-type ELISA. This method is based on G12 and A1 monoclonal antibodies (moAbs), which are known for their reactivity with the tandem epitopes present in the major GIP associated with CD.^2,21–24^ All samples were analyzed according to the manufactureŕs instructions. Each sample was analyzed in technical duplicates, and experiments were independently repeated using new tableware specimens, when used, for each temperature condition, contact time, and food sample, at least on two separate days, to ensure the accuracy and precision of the data. The GlutenTox G12-A1 ELISA Rapid Kit has a limit of detection (LoD) of 0.4 mg/kg and a limit of quantification (LoQ) of 1.2 mg/kg of gluten, which is why values below the calibration range are given as <LoQ.

### 2.5 Statistical Analysis

The results of the quantitative variables were expressed using the mean ± standards deviation (SD). Statistical analyses were performed using SPSS 25.0 (SPSS Inc., Chicago, IL, USA) and Graphpad Prism 10.4.2 for Windows. The goodness-of-fit to normality was calculated using the Shapiro–Wilk test. The Mann–Whitney *U* test was used to compare quantitative variables in independent groups, and the Wilcoxon signed-rank test was used to compare quantitative variables in dependent paired groups. The Friedman test was used to compare three or more related samples. Statistical analyses were performed using IBM SPSS Statistics 26.0 for Windows (IBM Corp., Armonk, NY, USA). Statistical significance was set at *p* < 0.05.

## 3 Results and Discussion

### 3.1 Baseline Gluten Analysis in Selected Food Samples

Before evaluating potential gluten transfer from biodegradable tableware to food, it was essential to verify the absence of gluten in the selected food items. To ensure that any detected gluten in subsequent experiments could be attributed solely to contact with the tableware, four commercially available food products, two solids (omelet and rice) and two liquids (vegetable cream and milk), were selected and analyzed for their baseline gluten content. All samples were labeled as gluten-free.

Omelet, a staple in the Spanish diet, provides a representative sample of a common dish frequently prepared in households, making it relevant for assessing real-world contamination risks.^25^ Rice, vegetable cream and milk, which are widely consumed in everyday diets for their nutritional value and are particularly valued for their versatility and naturally gluten-free nature. Additionally, these food items were selected because of their easy availability in supermarkets, ensuring their practical relevance and accessibility.

To confirm the absence of gluten, each sample was tested in duplicate directly from its original commercial packaging to avoid cross-contamination. The analysis revealed gluten concentrations below the LoQ, thus supporting the gluten-free claims of the selected food items and validating their suitability for subsequent transfer experiments.

### 3.2 Gluten Content in Biodegradable Tableware

To assess the potential risk of gluten transfer from biodegradable tableware to gluten-free foods, the gluten content of several commercially available items was analyzed using a sandwich ELISA. This method employed the G12 and A1 monoclonal antibodies, which are known to detect GIP and, therefore, provide a reliable measure of the immunogenic potential of gluten in complex matrices such as biodegradable materials.^26–28^ Among all tested samples, only one (dish 5) wheat-based dish exhibited detectable and significant levels of gluten, with a concentration of 48,486.7 ± 1760.2 mg/kg. In contrast, the remaining items, including those labeled as wheat-based and those made from other materials, showed gluten concentrations below the LoQ of the assay. These findings agree with prior studies conducted by the same research group, where high gluten levels exceeding the quantification range were detected in various biodegradable dishes.^29^ Similarly, a Dutch study reported gluten concentrations exceeding 40,000 mg/kg in wheat bran-based dishes and over 8000 mg/kg in edible organic straws.^30^

Interestingly, although several of the tested tableware products were primarily derived from wheat, only dish 5 contained gluten levels with a realistic risk of transfer to gluten-free food. This highlights the variability in gluten content among biodegradable products, even when manufactured from ostensibly similar raw materials. Such variability could be attributed not only to differences in manufacturing processes that either concentrate or mitigate gluten content, but also to the use of different plant parts (such as stalks, bran, or leaves rather than seeds), as well as to the application of additives, coatings, or other treatments during production.

The absence of quantifiable gluten in most samples suggests that, in many cases, manufacturing steps may effectively reduce gluten content to negligible levels. However, given the lack of transparency from manufacturers regarding these processes, further investigation is warranted. Understanding the mechanisms behind gluten reduction in these products could not only provide reassurance to individuals with GRDs but also inform regulatory agencies in developing appropriate safety guidelines for biodegradable materials intended for food contact.

### 3.3 Gluten Transfer to Food from Biodegradable Tableware at Room Temperature and after Heating

To rigorously evaluate the potential for gluten migration from biodegradable tableware items to food under realistic consumption conditions, this study investigated gluten transfer to a widely consumed liquid food (milk), considering both room temperature and controlled microwave heating of the packaging materials containing the food samples for varying periods of time. In this context, milk was selected as the test medium due to its widespread daily consumption in Mediterranean diets,^25^ as well as its physical properties as a liquid, which allow for full surface contact with the tableware, ensuring optimal exposure. This product was used to test all types of packaging materials (dishes, cups, and straws). The assay was performed carefully under controlled testing conditions. To closely replicate the actual eating process and account for mechanical actions that could influence gluten transfer, each food sample was continuously stirred using a metal spoon. This method was intended to simulate stirring and interactions that would typically occur during consumption. The application of this continuous was crucial for accurately mimicking real-world conditions and providing reliable data on gluten transfer.

Furthermore, to assess the effect of exposure on gluten migration, the samples were subjected either to room temperature conditions or to controlled microwave heating, maintaining a total exposure time of 30 min in both cases. The selected heating durations of 1, 2, and 3 min reflect typical domestic food preparation practices using standard household microwaves. These durations are commonly applied to reheat or prepare various foods and therefore represent realistic exposure scenarios. However, due to the evaporation properties of milk observed across all tableware types and the tendency of dish 5 to disintegrate and release fragments (Figure 2), gluten analysis was only feasible at room temperature and after 1 min of microwave heating, despite the labeling indicating that the container was microwave compatible.

**Figure 2:**
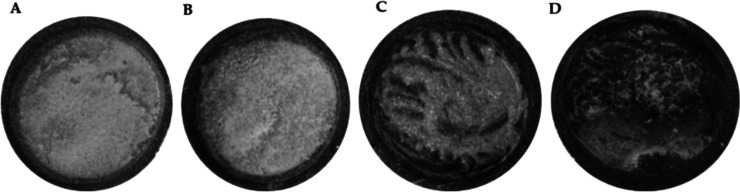
Physical alterations in milk assays when heated for different times (min) in a microwave. (A) Room temperature. (B) 1 min heated. (C) 2 min heated. (D) 3 min heated.

The findings of this study revealed that most values were below the <LoQ for gluten migration from the biodegradable samples to the liquids, except for those in contact with dish 5. These results were expected because dish 5 was the only dish that tested positive for gluten when analyzed individually. In contact with this tableware, the milk samples showed a content of 237.3 ± 12 mg/kg of gluten at room temperature and 479.1 ± 23.6 mg/kg when heated for 1 min. The results obtained indicated significantly high levels of gluten transfer, surpassing the legal claim threshold for gluten-free (<20 mg/kg) or low-gluten content products (<100 mg/kg).^11,12^ These findings suggest that while most biodegradable tableware appears safe under typical conditions, certain tableware items, depending on their composition or manufacturing processes, may pose a risk of significant gluten transfer. Thermal exposure may further exacerbate this migration. These results underscore the need for regulation and clearer labeling, especially for items derived from gluten-containing raw materials such as wheat.

### 3.4 Influence of Food Matrix on Gluten Migration from Biodegradable Tableware

To investigate the potential for gluten migration from biodegradable tableware, four representative gluten-free foods—previously confirmed to contain no detectable gluten—were selected: two solids (omelet and rice) and two liquids (vegetable cream and milk). These items were chosen due to their common consumption and differing characteristics, such as moisture content, viscosity, and potential for surface interaction. This variety may allow for a comprehensive assessment of how food properties, in combination with thermal and mechanical exposure, might influence gluten transfer from contaminated biodegradable materials. The analysis has the potential to provide valuable insight into the risk of gluten cross-contact, which may vary depending on the type of food and handling conditions.

Each food sample was brought into contact with dish 5, maintained at room temperature for 30 min. This contact period was chosen to simulate realistic food handling scenarios while ensuring sufficient exposure time for potential gluten migration to occur. Eating-like movements were performed consistently to replicate typical usage. The quantification of gluten content revealed varying levels of transfer among the different food types, with most values exceeding the 20 mg/kg threshold. Measured gluten concentrations were 23.2 ± 4.1; 11.2 ± 0.1; 2107.2 ± 217.9; and 237.3 ± 12.0 mg/kg for omelet, rice, vegetable cream, and milk, respectively (Figure 3). Rice showed the lowest level of gluten transfer, with concentrations remaining below the established threshold, highlighting how food composition may influence the extent of gluten migration.

**Figure 3:**
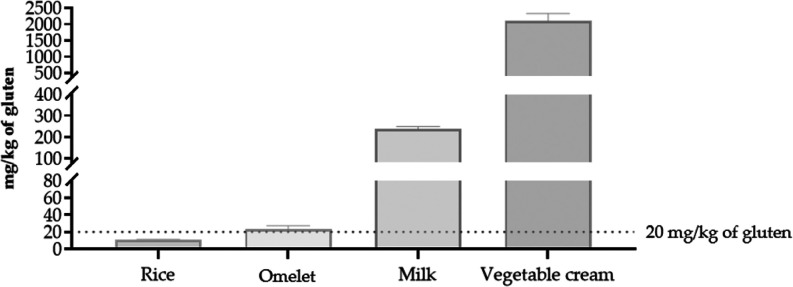
Gluten content in gluten-free foods after 30 min of contact at room temperature with a wheat-based biodegradable dish. Results were expressed as mg/kg of gluten (mean ± SD). SD, standard deviation.

The degree of gluten transfer was strongly influenced by the physical state and specific characteristics of each food item, reinforcing the importance of considering food consistency and matrix composition in gluten migration studies. The data revealed a pronounced disparity between solid and liquid foods, with liquid matrices, namely milk and vegetable creams, showing significantly higher levels of gluten transfer than solids such as rice and omelet (Mann–Whitney test, *p* < 0.001). These findings highlight the greater susceptibility of liquid foods to gluten cross-contact, likely due to their higher moisture content, lower viscosity, and greater surface spread, which facilitate more extensive interaction with the contaminated biodegradable material. Interestingly, significant differences were also observed between the two liquid samples. Vegetable cream showed the highest gluten concentration (2107.2 ± 217.9 mg/kg), far exceeding that of milk (237.3 ± 12.0 mg/kg). This disparity may be attributed to differences in fat content, emulsion stability, or other compositional factors that modulate the interaction with the tableware surface and the potential for gluten adherence and migration.

Taken together, these results underscore the critical role of food consistency and composition in gluten cross-contact events. Liquid foods, particularly those with complex or emulsified structures, not only exhibit a higher propensity for gluten transfer but may also induce material changes that further facilitate gluten mobility and retention. These findings are especially relevant for individuals with GRDs, where even minimal gluten exposure can have serious health implications. The study emphasizes the need for enhanced food safety protocols and stricter oversight in scenarios where biodegradable tableware is used in combination with high-risk food types, particularly liquid products, to prevent unintentional gluten exposure.

### 3.5 Effect of Contact Time on Gluten Transfer to Food from Contaminated Biodegradable Tableware at Room Temperature

To characterize gluten migration dynamics, the effect of food-surface contact time on gluten transfer was evaluated. This variable was selected based on preliminary results showing significant variability in gluten transfer across different food matrices. To simulate typical food handling and consumption conditions, contact times of 5, 10, 15, and 30 min were established. This approach allowed for the examination of how specific physicochemical properties of foods, such as moisture content, viscosity, and surface adhesion, may influence their ability to absorb gluten from contaminated biodegradable materials.

As for the solid foods (omelet and rice), gluten transfer from dish into omelet showed the following concentrations: 9.6 ± 1.7; 29.3 ± 2.8; 24.6 ± 0.4; and 23.2 ± 4.2 mg/kg at 5, 10, 15, and 30 min, respectively. The results of rice sample were: 11.2 ± 0.3; 16.7 ± 2.9; 8.5 ± 0.1 and 11.2 ± 0.1 mg/kg at 5, 10, 15, and 30 min, respectively (Figure 4A,B). For omelet, however, although gluten transfer remained relatively stable over time, a peak concentration was observed at 10 min (29.3 ± 2.8 mg/kg), exceeding the 20 mg/kg threshold. Subsequently, gluten levels slightly decreased at 15 and 30 min but remained above the threshold. This is of particular concern, as it exceeds the 20 mg/kg limit set by many regulatory standards for gluten-free foods.^12^ This pattern suggests that gluten migration into solid foods like omelet may occur rapidly within the first few min of contact and not necessarily increase progressively with longer exposure times. Interestingly, rice consistently exhibited lower levels of gluten transfer compared to the omelet, suggesting that the type and texture of the food matrix may play a critical role in gluten retention. Although the overall trend indicates a minimal impact of contact time on gluten transfer in solid foods, the elevated gluten concentration observed in the omelet after 10 min of exposure raises concern about a potential increase in risk with prolonged contact. Moreover, the fluctuations in gluten concentrations over time, rather than a linear or progressive increase, suggest that gluten transfer in solid foods may be influenced by complex, nonlinear interactions between the food matrix and the contact surface, rather than by exposure duration alone. Together, these findings underscore the importance of considering both the physical characteristics of the food and the duration of exposure when assessing cross-contact risks in gluten-free food preparation.

**Figure 4:**
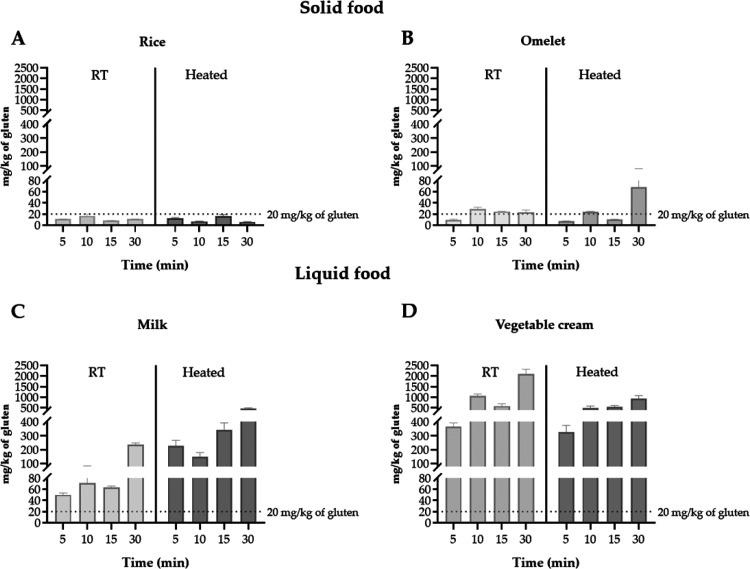
Gluten determination in gluten-free foods after exposure to two different temperature conditions and increasing contact times with wheat-based dish. (A) Rice. (B) Omelet. (C) Milk. (D) Vegetable cream. Results were expressed as mg/kg of gluten (mean ± SD). SD, standard deviation; RT, room temperature.

To further explore the dynamics of gluten transfer, the same experimental procedure was subsequently applied to liquid food matrices. The outcomes revealed notable differences between vegetable cream and milk. Specifically, gluten concentrations in vegetable cream at 5, 10, 15, and 30 min were 365.2 ± 27.1; 1060.2 ± 87.7; 590.9 ± 104.1 and 2107.2 ± 217.9 mg/kg, respectively. In contrast, milk samples exhibited lower concentrations of 50.1 ± 3.4; 71.2 ± 11.9; 63.9 ± 2.0; and 237.3 ± 12.0 mg/kg over the same time intervals (Figure 4C,D). The results suggest a time-dependent increase in gluten transfer. While a Friedman test indicated that this trend was not statistically significant (*p* = 0.112), the lack of significance is likely attributable to the limited sample size, rather than the absence of a real effect. The observed patterns support the hypothesis that the physical properties of the liquid matrix, particularly viscosity or/and density, play a critical role in mediating gluten migration. Vegetable cream, being more viscous and cohesive, may facilitate greater gluten retention or adsorption compared to the relatively fluid milk matrix. Of particular concern is the fact that all vegetable cream samples, including those with only 5 min of exposure, exceeded the 100 mg/kg gluten concentration threshold (Figure 4B). This is noteworthy from a regulatory standpoint, as 100 mg/kg is often cited in international legislative norms as the upper boundary foods labeled as “low gluten”.^12^ Such findings raise critical questions about the adequacy of regulations, which predominantly focus on gluten levels in final products, but do not explicitly address cross-contact or dynamics of the gluten transfer during processing or storage. Indeed, the European Commission regulation No. 1935/2004, in its third article, states: “Any material or article intended to come into contact directly or indirectly with food must be sufficiently inert to preclude substances from being transferred to food in quantities large enough to endanger human health or to bring about an unacceptable change in the composition of the food or a deterioration in its organoleptic properties of the food”.^31^ However, our data demonstrate that this requirement may not be fulfilled in real-world scenarios involving individuals with gluten sensitivity or CD, particularly when using materials intended for food contact under conditions that simulate common food-handling practices.

The progressive increase in gluten contamination over time highlights the dynamic and cumulative nature of gluten transfer in liquid foods. This underscores the necessity of incorporating the physicochemical characteristics of foods, such as viscosity, into contamination risk assessments. The substantial increase in vegetable cream implies that more viscous liquids may facilitate greater gluten transfer than liquids such as milk. Moreover, our study identified considerable variability in gluten quantification between replicate samples even under strictly controlled conditions of temperature and exposure time. Collectively, these findings emphasize that even short-term exposure can result in measurable gluten transfer, highlighting the need for stringent cross-contact prevention strategies. Such measures are critical not only in industrial food processing environments but also in domestic and institutional settings, where individuals with GRDs are most vulnerable.

### 3.6 Effect of Temperature on Gluten Transfer to Food from Contaminated Biodegradable Tableware

Given its relevance to everyday food preparation, temperature was selected as a variable for analysis. Two temperature conditions were examined: room temperature and 1 min microwave heating, with the heating time included as part of the total contact time. Exposure times were tested at increasing intervals of 5, 10, 15, and 30 min, consistent with the previous assay.

To assess the influence of heat on gluten transfer, gluten concentrations were measured in each food item after microwave heating. In rice, levels were 12.6 ± 0.8; 7.0 ± 0.5; 16.5 ± 2.7 and 6 ± 0.3 mg/kg at 5, 10, 15, and 30 min, respectively. The heated omelet showed more variable concentrations of 7.2 ± 0.4; 24.4 ± 0.6; 10.1 ± 0.9 and 68.6 ± 12.8 mg/kg over the same time points. In contrast, significantly higher gluten migration was observed in liquid foods. Milk samples contained 227.6 ± 39.5; 149.7 ± 29.1; 342.3 ± 49.4 and 479.1 ± 23.6 mg/kg, while vegetable cream reached the highest levels: 324.9 ± 49.3; 492.4 ± 98.4; 553.1 ± 60.8 and 933.9 ± 144.2 mg/kg, respectively. These results underscore the significant influence of both the food matrix and contact time on the extent of gluten transfer under heat exposure.

When comparing the gluten levels of heated samples with those of their room temperature counterparts exposed for the same duration, unexpectedly lower levels of gluten were detected in some of the heated samples. After 10 min of total exposure, the gluten concentration in rice was 16.7 ± 2.9 mg/kg at room temperature, compared to 7.0 ± 0.5 mg/kg when the sample had been briefly heated at the beginning of the exposure. After 30 min, the concentrations were 11.2 ± 0.1 mg/kg at room temperature and 6.0 ± 0.3 mg/kg after 1 min of heating (Figure 4A). A similar trend was observed in the omelet samples. At 5 min, the gluten content at room temperature was 9.6 ± 1.7 mg/kg, while it was 7.2 ± 0.4 mg/kg after 1 min of heating. At 15 min, the gluten concentration was 24.6 ± 0.4 mg/kg at room temperature and 10.1 ± 0.9 mg/kg after 1 min of heating, respectively (Figure 4B). Notably, milk was the only food that showed an increase in gluten content after the heat treatment (Figure 4C). However, all vegetable cream samples showed decreased gluten content at all contact times (Figure 4D).

The results of this study provide intriguing insights into the effects of heating on gluten transfer from contaminated biodegradable tableware to food. The observed trend of lower gluten levels in heated samples compared to their room temperature counterparts at equivalent exposure times was unexpected and warrants further exploration. One potential explanation for this phenomenon could be the denaturation or partial degradation of gluten proteins upon brief exposure to heat, particularly in the context of certain food matrices like omelet and rice. Heating may lead to conformational changes in gluten proteins, altering their solubility or reducing their ability to adhere to the surface of the food, thus resulting in lower gluten absorption from the contaminated tableware. This pattern suggests that heat may disrupt the transfer of gluten into these food matrices, possibly due to changes in the texture or physical properties of the food. For example, the protein denaturation and moisture evaporation during heating could affect the gluten-binding capacity of the food, leading to reduced gluten retention.

Interestingly, milk was the only food tested that showed an increase in gluten content following the heat treatment. This result suggests that the physical properties of milk, such as its relatively low viscosity, may facilitate the dissolution or migration of gluten from the contaminated surface, particularly when exposed to heat. It is possible that the heating process increases the solubility of gluten in milk, leading to a higher gluten transfer.

Overall, the findings suggest that heating can influence gluten transfer dynamics in a complex manner, with the nature of the food matrix playing a critical role. The lower gluten levels observed in some heated samples may be due to protein denaturation or altered binding capacities, while the increase in gluten transfer observed in milk highlights the need to consider both food properties and heat exposure when assessing cross-contact risks in food preparation. Future studies are needed to further elucidate the specific mechanisms underlying these observations, including the role of different heating methods, durations, and the physicochemical characteristics of various food matrices in gluten transfer.

This study demonstrates that certain biodegradable materials intended for food contact, particularly those derived from wheat, may pose an unrecognized risk of gluten contamination in gluten-free foods. Our results confirm that gluten migration can occur under common consumption conditions in both solid and liquid food matrices. Liquid and emulsified foods, such as vegetable cream and milk, showed a notably higher susceptibility to gluten transfer, with levels far exceeding the 20 mg/kg threshold established for gluten-free labeling.

A marked variability in gluten content was observed among similarly labeled biodegradable items, revealing a lack of transparency regarding raw materials and manufacturing processes. The findings highlight the critical influence of food matrix, contact time, and temperature on gluten migration dynamics, factors currently overlooked by existing food packaging regulations.

Given the growing use of biodegradable materials, these results underscore the urgent need for regulatory oversight and mandatory allergen labeling for food-contact materials. Stricter safety standards and improved traceability in manufacturing practices are essential to protect individuals with GRDs and preserve the integrity of the GFD. Moreover, as shown in Figure 2, the swelling (and partial disintegration) of wheat-based dish 5 upon contact with liquid foods indicates noncompliance with the inertness requirement for food contact materials. From a food safety perspective, this structural change is critical, as it shows that this material is unsuitable for use with heated liquid foods.

Although this study focused on gluten, the potential migration of other food allergens, such as milk, egg, soy, or nut proteins, should not be ruled out, especially in biodegradable materials derived from allergenic sources. Future research should aim to assess the presence and transferability of multiple allergens in these materials and across diverse food matrices. A broader allergen risk assessment framework would support the development of comprehensive safety standards to protect all food-allergic or food-sensitive consumers.

## Supplementary Material



## Notes

Table S1: Information on foods composition (PDF)

## Abbreviations

CD: celiac disease
ELISA: enzyme-linked immunosorbent assay
EU: European Union
GFD: gluten-free diet
GIP: gluten immunogenic peptides
GRDs: gluten-related disorders
LoQ: limit of quantification
moAbs: monoclonal antibodies
NCGS: non-celiac gluten sensitivity
SD: standard deviation
SPSS: statistical package for the social sciences
UGES: universal gluten extraction solution
