# Supplementary material for: Potential Transfer of Toxic Gluten from Biodegradable Tableware to Gluten-Free Foods: Implications for Individuals with Gluten-Related Disorders
Source: J Agric Food Chem. 2025 Oct 22;73(44):28386–94. doi: 10.1021/acs.jafc.5c07516 (PMC12593404; doi:10.1021/acs.jafc.5c07516)
Supplement: Supplementary file 1 [file jf5c07516_si_001.pdf]

Supporting Information

Potential Transfer of Toxic Gluten from Biodegradable Tableware to Gluten-Free Foods: Implications for Individuals with Gluten-Related Disorders

Carolina Sousa[1], Abel Heredia[1], Lucía de Arcos[1], Verónica Segura[1], Ángela Ruiz-Carnicer[1 * †], Isabel Comino[1 * †]

[1] Department of Microbiology and Parasitology, Faculty of Pharmacy, University of Seville, 41012 Seville, Spain; csoumar@us.es (C.S.); ahbarroso@us.es (A.H); lucdedom@alum.us.es (L.A); vsegura@us.es (V.S.); acarnicer@us.es (Á.R.-C.); icomino@us.es (I.C.)

[†] These authors contributed equally to this work and share first authorship.

[*] Correspondence: acarnicer@us.es; icomino@us.es; Tel.: +34-954-556-452

Supplementary Table 1.

Food matrix	Food	Composition per 100 g or 100 mL				
		Fats	Carbohydrates	Proteins	Fiber	Salt
**Solid foods**	Rice	3.4	49.6	3.7	1.2	1
	Omelet	11	12	6	2	1
**Liquid foods**	Milk	3.6	4.6	3.1	0	0.13
	Vegetable cream	2.2	4.6	0.7	1.1	0.68
